# Development of a Real-Time Quantitative PCR Based on a TaqMan-MGB Probe for the Rapid Detection of *Theileria haneyi*

**DOI:** 10.3390/microorganisms11112633

**Published:** 2023-10-26

**Authors:** Bingqian Zhou, Guangpu Yang, Zhe Hu, Kewei Chen, Wei Guo, Xiaojun Wang, Cheng Du

**Affiliations:** State Key Laboratory of Animal Disease Control and Prevention, Harbin Veterinary Research Institute, Chinese Academy of Agricultural Sciences, Harbin 150069, China; zhoubingqian9901@163.com (B.Z.); yangguangpu183@163.com (G.Y.); huzhe@caas.cn (Z.H.); 18838970816@163.com (K.C.); guowei@caas.cn (W.G.)

**Keywords:** *Theileria haneyi*, real-time quantitative PCR, nested PCR

## Abstract

Equine piroplasmosis (EP) is a parasitic disease caused by *Theileria equi* (*T. equi*), *Babesia caballi* (*B. caballi*) and *Theileria haneyi* (*T. haneyi*). This disease is considered to be reportable by the World Organization for Animal Health (WOAH). Real-time quantitative PCR (qPCR) is regarded as a straightforward, rapid and sensitive diagnostic method to detect pathogens. However, qPCR has not been employed in the various epidemiological investigations of *T. haneyi*. In this study, we developed a new qPCR method to detect *T. haneyi* based on the chr1sco (chromosome 1 single-copy open reading frame (ORF)) gene, which has no detectable orthologs in *T. equi* or *B. caballi.* A TaqMan MGB probe was used in the development of the qPCR assay. A plasmid containing the chr1sco gene was constructed and used to establish the standard curves. The novel qPCR technique demonstrated great specificity for detecting additional frequent equine infectious pathogens and sensitivity for detecting diluted standard plasmids. This qPCR was further validated by comparison with an optimized nested PCR (nPCR) assay in the analysis of 96 clinical samples. The agreement between the nPCR assay and the established qPCR assay was 85.42%. The newly established method could contribute to the accurate diagnosis of *T. haneyi* infections in horses.

## 1. Introduction

Equine piroplasmosis (EP) is a tick-borne disease caused by the intraerythrocytic protozoan parasites *Theileria equi* (*T. equi*), *Babesia caballi* (*B. caballi*) and *Theileria haneyi* (*T. haneyi*) [1], in several equid species, including horses, mules, donkeys and zebras. Piroplasmosis is a globally significant disease [2] and causes significant economic losses in the equine industry around the world, with the primary economic importance being the impact on the international export of horses for participation in equestrian sporting events [3]. EP is considered to be a reportable disease by the World Organization for Animal Health (WOAH). Data from The Food and Agricultural Organization of the United Nations suggests that approximately 90% of the global horse population resides in EP-endemic areas [3]. EP is common in subtropical and tropical areas of the world and is known to be endemic in several countries in Africa, Asia, the Americas and Europe [4,5,6]. The disease can cause mild subclinical to severe acute clinical signs. The peracute form of EP has only been observed in dead or dying animals, while acute and chronic infections are characterized by variable clinical symptoms, including mild inappetence with anemia, fever, inappetence, splenomegaly and oedema [7]. EP is transmitted to vertebrate hosts by several species of ixodid ticks. Ticks are obligate hematophagous ectoparasites that negatively impact humans, domestic and wild animals directly through tick bites and blood feeding, and indirectly by transmission of viral, bacterial, and protozoan pathogens [8]. Tick transmissions are diverse and uncontrollable; hence, EP has a big impact on long-distance trans-regional spread. *T. equi* and *B. caballi* have been recognized as the species that cause EP and they belong to the phylum Apicomplexa and the order Piroplasmida [9]. In 2018, a study in Texas, USA suggested that *T. haneyi* in the same order was another potential EP agent [10]. The shape of *T. haneyi* is the same as that of *T. equi*, although *T. haneyi* is smaller, having a mean length of 1.15 ± 0.16 µm and a width of 0.70 ± 0.07 µm [10]. The equimerozoite antigens (EMAs), termed merozoite surface antigens are surface-exposed proteins, expressed during the infection of these parasites. Because they are immunodominant and conserved diagnostic antigens of *T. equi*, members of EMAs have drawn considerable interest from the veterinary diagnostic community [11,12]. Both *T. equi* and *T. haneyi* contain the nine members of the EMA superfamily, which were previously found as a multigene family only in *T. equi*. Interestingly, although ema1, 3 and 4 genes are absent from the *T. haneyi* genome, three novel EMA family members, ema11-13 have been found in *T. haneyi* [13]. *T. haneyi* causes milder clinical disease than *T. equi* in experimentally infected horses and is capable of co-infection with *T. equi*. Most equine *T. equi* infections are treated with the antiparasitic medication imidocarb diproprionate (ID), but *T. haneyi* is not susceptible to ID due to genomic differences. Previous studies have discovered that horses in the Gambia, Nigeria and South Africa are superinfected with *T. equi* and *T. haneyi* or triple infected with *T. equi*, *T. haneyi* and *B. caballi* [14,15]. Therefore, the accurate diagnosis of *T. haneyi* is needed to implement the correct treatment and to prevent further disease.

Before horses are transported internationally, an EP evaluation is required. Clinical symptoms, such as anemia, fever, inappetence, splenomegaly and oedema, can be used to make a preliminary diagnosis of the illness during detection, but laboratory testing is ultimately necessary to confirm the diagnosis, particularly when the illness is in its incubation or prodromal stages. Laboratory diagnosis mainly relies on molecular biology and immunological detection methods. A number of detection methods have been developed for the diagnosis of *B. caballi* and *T. equi*, including microscopic examination, culture, serology and molecular assays [2]. However, few detection methods are available for the diagnosis of *T. haneyi*. An indirect ELISA has been developed to identify *T. haneyi* antibodies in equids based on EMA11, and this technique may identify antibodies in equine serum samples from various geographic locations [13]. However, the serological tests still detect specific antibodies for weeks or months after successful treatment and there is also a risk of false-positive results due to cross-reactivity with antibodies caused by other infections. Because of the challenges associated with serological tests, molecular tests have been the diagnostic assays of choice for the detection of infection with certain causal agents. For parasitological diagnosis, molecular tests are very specific, because they can detect parasite-specific DNA or RNA, and molecular assays with high sensitivity and low cost might enable the detection of *T. haneyi* nucleic acids. It has been reported that nested PCR (nPCR) assays have helped to overcome the challenges in detecting the low parasitaemia found in *T. haneyi* infections [10]. However, other PCR assays, including real-time quantitative PCR (qPCR), have not been employed in epidemiological investigations of *T. haneyi* to date, and the limited number of molecular detection methods available for *T. haneyi* hinders widespread assessment of the prevalence of this recently discovered organism.

qPCR is a straightforward, rapid and sensitive diagnostic tool to detect pathogens. Previous reports have reported qPCRs based on 18S rRNA that can be used to detect *T. equi* and *B. caballi* [16]. However, there is no qPCR currently available for the detection of *T. haneyi*. The two types of qPCR assays that have been most frequently used in pathogen detection are those with probe-based chemistry (e.g., TaqMan probes or scorpion probes), and those using dsDNA binding dyes (e.g., SYBRGreen, PicoGreen or EvaGreen) [17]. Assays using dsDNA binding dyes are relatively cost-effective and are easy to use. However, they offer lower specificity than the probe-based method because the dsDNA-binding dye is non-specific. The probe-based qPCR assay requires the binding of primers and probes to the DNA sample for the fluorescent dyes to become observable. This is advisable when diagnosing pathogens, because it reduces the fluorescence background caused by nonspecific PCR products and, therefore, improves the sensitivity of the assay [18,19].

**Figure 1:**
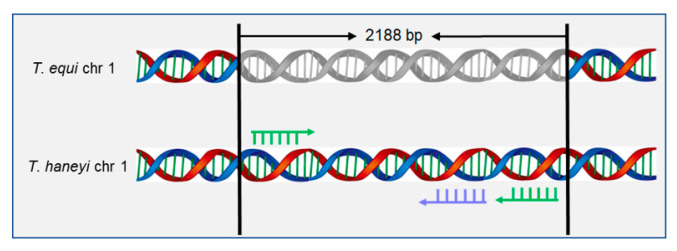
Schematic diagrams of the chromosome 1 single-copy open reading frame (chr1sco) in the *T. equi* and *T. haneyi* genomes. The double helix structure of *T. equi* and *T. haneyi* gene was colored with blue and red. The primers used in this study were labeled with green arrows and the probe used in this study was labeled with purple arrow.

Genomic analysis has revealed that there is an overall genomic divergence between *T. equi* and *T. haneyi*, and the genome of *T. equi* is approximately 2 Mbp larger than that of *T. haneyi*. Interestingly, sequencing of the *T. haneyi* genome revealed the absence of specific EMA family members, and it has been reported that the EMA genes ema1, ema3 and ema4, all located on chromosome 1 single-copy open reading frame (chr1sco) in *T. equi*, have no detectable orthologs in *T. haneyi*. However, *T. haneyi* contains three distinct EMA genes, named ema11-13, two of which are located on chromosome 1 and one on chromosome 2. Through the direct bacterial artificial chromosome (BAC) sequencing verification with standard dideoxy sequencing, a previous study found that a 2118 bp single-copy chr1sco of *T. haneyi* does not have a homolog in *T. equi* (Figure 1). Based on this, the researchers established the nPCR assay to detect the new potential species of EP [10]. The chr1sco specific to the *T. haneyi* genome can, therefore, be used to improve the accuracy of *T. haneyi* infection diagnosis in horses.

In this study, the chr1sco gene sequence specific to *T. haneyi* was used as a detection target. We designed qPCR primers for *T. haneyi* and established a qPCR based on a TaqMan-MGB probe assay for the detection of *T. haneyi*. The specificity and sensitivity of the method were evaluated, and the assay was validated through a comparison of the newly developed qPCR method with the optimized nPCR method, in the evaluation of 96 clinical samples stored in our laboratory. In summary, this newly developed qPCR assay will be helpful for detecting EP caused by *T. haneyi* and controlling the epidemic.

## 2. Materials and Methods

### 2.1. Sample Collection and DNA Extraction

Equine blood samples positive for other equine pathogens, including equine herpesvirus-1 (EHV-1), equine herpesvirus-4 (EHV-4), *Salmonella abortus equi* (*S. abortus equi*), *Salmonella Dublin* (*S. Dublin*), *Salmonella typhimurium* (*S. typhimurium*), *T. equi*, *B. caballi* and *Salmonella enteritidis*, isolated from horses by our laboratory were used to test the specificity of our novel detection method. The positive horse blood sample for *T. haneyi* was collected from a horse with clinical symptoms of EP. A serological test was used to detect the antibodies in the blood sample and the result showed that this infected horse produced high-level antibodies. Before *T. haneyi* was identified and obtention of the positive sample, we excluded samples with infections of *B. caballi* or *T. equi* using reported PCR [20]. The sample that was positive for *T. haneyi* but negative for *T. equi* or *B. caballi* was amplified using the nPCR with chr1sco primers developed by researchers who first found this pathogen in 2018 [10]. This sample was sequenced, and the sequence of the amplicon generated from this sample was blasted unambiguously with the sequence data downloaded from GenBank (*T. haneyi*, MT896770.1). The negative blood sample was detected using the serological test and the reported nPCR of *T. haneyi* [10]. After collection into K2-EDTA vacuum tubes (Solarbio, Beijing, China), all pathogen samples were stored at −20 °C until use. Genomic DNA was extracted from each 200 μL sample using a blood DNA extraction kit (TIANGEN, Beijing, China) according to the recommendations of the manufacturer, and was stored at −20 °C until use.

### 2.2. Selection for Specific Primers and Probes

Primers for the detection of *T. haneyi* were designed in Primer Premier 5.0 based on the sequence of chr1sco from *T. haneyi* in GenBank (*T. haneyi*, MT896770.1) (Table 1). The chr1sco, which does not have a homolog in *T. equi*, is a 2118 bp fragment and is specific to the *T. haneyi* genome [10]. The 5′ end and 3′ end of the TaqMan probe used in this assay was differently labeled with the FAM fluorophore and the Minor Groove Binder (MGB), respectively, according to the manufacturer’s guidelines (Thermo Scientific, Waltham, MA, USA) (Table 1). To check that the primers and probe were specific to the *T. haneyi* chr1sco, the chr1sco gene was amplified and cloned into pMD-18T plasmids (TaKaRa, Kyoto, Japan).

### 2.3. Plasmid Construction and Standard Curve Generation

After transformation into chemically competent DH5α cells following the manufacturer’s instructions, the recombinant plasmids were extracted using the Fast Pure Plasmid Mini Kit from Vazyme (Nanjing, China). A spectrophotometer (Berthold, Wildbad, Germany) was then used to measure the OD_260_ in order to quantify the concentration of the plasmids and the number of copies was calculated using the following formula: y (copies/μL) = (6.02 × 10^23^) × (x (ng/μL) × 10^−9^ DNA)/(DNA length × 660) [21]. The standard plasmid was serially diluted 10-fold with ddH_2_O and amplified using the optimized qPCR system at a concentration of 1.0 × 10^8^–1.0 × 10^2^ copies/μL. The final standard curve was generated based on the CT value and the logarithm of the standard copy number.

### 2.4. Real-Time Quantitative PCR

Premix Ex Taq (Probe qPCR, 2×) (TaKaRa, Kyoto, Japan) and a qPCR System Mx3000P (Stratagene, La Jolla, CA, USA) were used in this qPCR. The concentrations of primers and probe of this qPCR were adjusted to achieve the largest cycle-to-cycle rise (ΔRn) and lowest threshold cycle (Ct) for each individual fluorescent signal. A 25 μL duplex solution mixture containing 2 μL of sample DNA or standard DNA, 12.5 μL of Premix Ex Taq, 1 μL of each pair of *T. haneyi*-F and *T. haneyi*-R (Table 1) (10 μM), and 1 μL of *T. haneyi*-Probe (Table 1) (10 μM) was used to conduct the qPCR reaction. Then, 7.5 μL nuclease-free water was added to a final volume of 25 µL. We optimized reaction conditions, including the different annealing temperatures and the number of cycles. Then, amplification was carried out using the following thermal cycling conditions: initial denaturation at 95 °C for 30 s, and 40 cycles of thermocycling, including denaturation at 95 °C for 5 s and annealing and extension at 60 °C for 30 s each. The real-time fluorescence values were assessed at the end of each annealing step. After testing each sample, the Ct value was determined using the log-linear phase of each reaction. All reactions included positive and negative controls.

### 2.5. Nested PCR Amplification of the Chr1sco of T. haneyi

A previous study designed an nPCR for the detection of *T. haneyi* based on the chr1sco [10]. The primers were the same as described previously, but we optimized the process of the nPCR. For the first round, 10 μL Taq PCR StarMix (GenStar, Beijing, China), 1 μL of each external primer, 2 μL of genomic DNA, and 6 μL of ddH_2_O were used in 20 μL of final volume for the reactions. An initial cycle of 98 °C for 2 min was used to start the PCR amplification process. This was followed by 30 cycles of 95 °C for 15 s, 57 °C for 30 s and 72 °C for 2 min, with a final extension of 72 °C for 8 min. A total of 2 μL of each PCR product from the first round of amplification served as the template for the second cycle, which used internal primers. The amplification was the same as the first round, but the number of cycles was changed to 35. Positive and negative controls were included for each reaction.

### 2.6. Sensitivity and Specificity

The sensitivity of the new qPCR assay in the detection of *T. haneyi* was assessed in 10-fold serially diluted standard plasmids (10^8^ to 10^1^ copies/μL). To evaluate the specificity of this assay, the nucleic acids of *T. haneyi* as well as other common pathogens of equine, including *S. Dublin*, *S. typhimurium*, EHV-4, EHV-1, *S. abortus equi*, *T. equi*, *B. caballi* and *Salmonella enteritidis*, were tested, and the horse blood DNA negative for *T. haneyi* was used as a negative control. The horse blood DNA positive for *T. haneyi* was used as the positive control.

## 3. Results

### 3.1. Standard Curve for the Real-Time Quantitative PCR Assay

**Figure 2:**
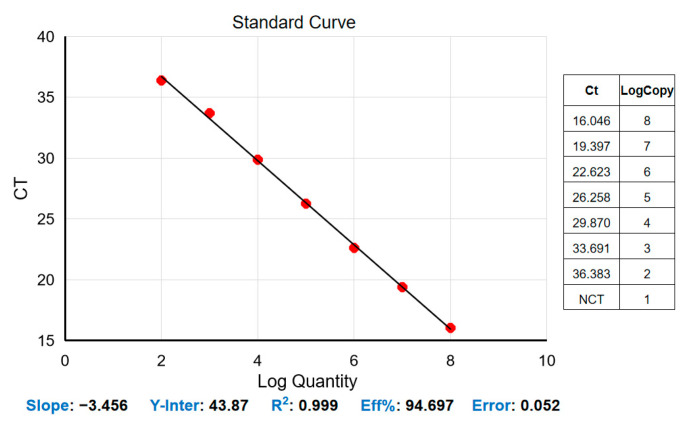
Standard curve of the real-time fluorescence of the quantitative PCR assay. The quantified DNA plasmid (10^8^–10^2^ copies/μL), employed as a positive control, was diluted three times in 10-fold serial dilutions, and the examination of these three replicates produced the curve.

The construction of a standard curve with the logarithm of the DNA copy number and the obtained CT values was tested using serial dilutions of standard plasmid DNA at concentrations between 1 × 10^8^ and 1 × 10^2^ copies/μL. The correlation coefficient R^2^ was found to be 0.999, the slope of the line was −3.456, and the amplification efficiency (Eff%) was 94.697%. The standard formula for *T. haneyi* is y = −3.456x + 43.87 (Figure 2). The newly developed qPCR assay showed high efficiency in detecting standard plasmids of *T. haneyi* DNA.

### 3.2. Analytical Sensitivity of the Real-Time Quantitative PCR Assay

**Figure 3:**
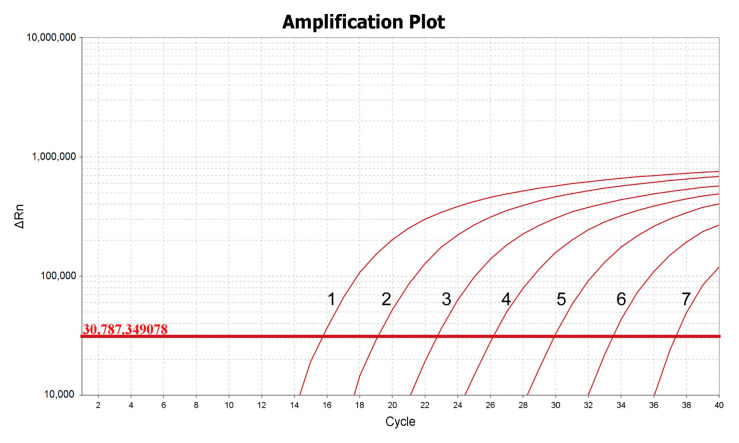
Sensitivity of the qPCR assay based on the detection of 10-fold serial dilutions of the standard plasmid of *T. haneyi*. The line 1–7 were symbolled the concentration of 1.0 × 10^8^–1.0 × 10^2^ copies/μL.

To determine the sensitivity of the qPCR method for detecting *T. haneyi*, the plasmid carrying full-length of *T. haneyi* chr1sco was subjected to a 10-fold serial dilution with TE buffer to create a dilution series. The dilutions were then used separately as the templates for qPCR amplification. The minimum detection limit of the new qPCR assay was found to be 1 × 10^2^ copies/μL (Figure 3), and the minimum detection limit of the optimized nPCR was 5.4 ng/µL. These results demonstrated the high sensitivity of this qPCR method.

### 3.3. Specificity of the Real-Time Quantitative PCR Assay

**Figure 4:**
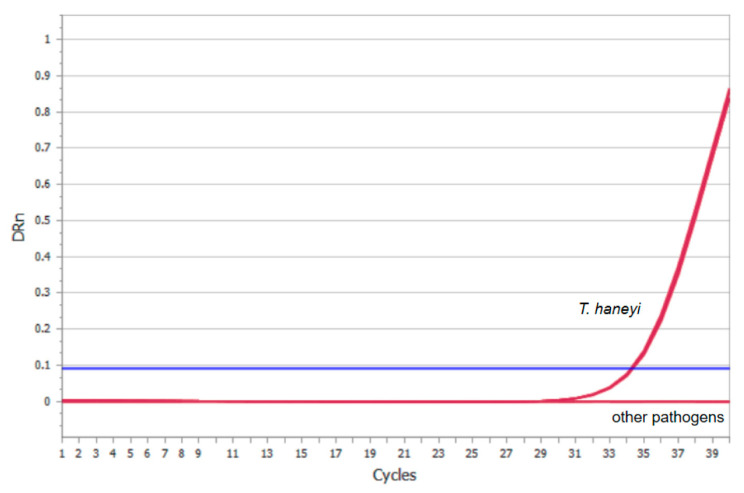
Specificity of the qPCR assay. The DNAs or cDNAs of other unrelated equine pathogens were used as templates. Other pathogens included *S. Dublin*, *S. typhimurium*, EHV-4, EHV-1, *S1. abortus equi*, *T. equi*, *B. caballi* and *Salmonella enteritidis*. The samples of pathogens were labeled with red lines and the blue line was the stander of negative sample.

Specific primers and a TaqMan probe were designed for the target gene of *T. haneyi*. We then assessed the specificity of our system. When the positive clinical samples of *T. haneyi* were utilized as templates in our recently developed qPCR test, a FAM fluorescent signal could be detected. The results of the qPCR panel analytical specificity studies are shown in Figure 4. For the negative control and other unrelated equine pathogens (*S. Dublin*, *S. typhimurium*, EHV-4, EHV-1, *S1. abortus equi*, *T. equi*, *B. caballi*, *Salmonella enteritidis*), the assay gave no signal. These findings supported the high specificity of this method.

### 3.4. Clinical Performance

**Figure 5:**
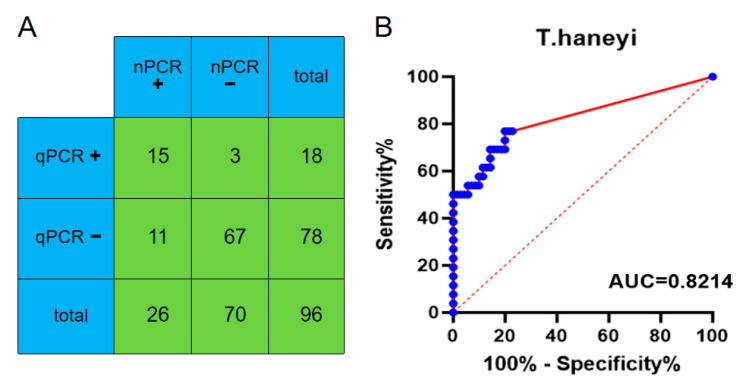
Comparison of the results from the nPCR and the new qPCR when testing the clinical samples. (**A**) The agreement between the nPCR and the new qPCR. The positive samples were labeled with + and the negative samples were labeled with −. (**B**) The ROC curve was plotted by calculating the diagnostic sensitivity and specificity of the new qPCR CT values relative to the results of the nested PCR assay. The clinical samples were labeled with the blue pots and the area under the red line represents the area under the ROC curve.

To further verify the clinical potential of this qPCR assay by comparison with the optimized nPCR assay, we tested 96 clinical samples using both of these two methods. The newly established qPCR assay identified 18 samples as *T. haneyi* positive. The nPCR assay identified 26 samples as *T. haneyi* positive. The agreement between the nPCR assay and the established qPCR assay was 85.42% (Figure 5). Compared with the nPCR method, the AUCs of the qPCR assay was 0.8214 for *T. haneyi* (Figure 5).

## 4. Discussion

EP is a tick-borne disease caused by *T. equi*, *B. caballi* and *T. haneyi* that has caused tremendous economic losses in the equine industry. *T. haneyi*, which was discovered in 2018, appears to have a global distribution, with infections seen in North America, South America and Africa [16,22]. Because of the insusceptibility of *T. haneyi* to ID, co-infection of *T. haneyi* and *T. equi* may interfere with the ID clearance of *T. equi* [23], and the presence of *T. haneyi* in equids makes the effective prevention and control of EP very complicated. For the treatment and prevention of the spread of EP, quick *T. haneyi* detection and identification are essential. Clinical symptoms can be utilized to form a preliminary diagnosis of the illness during detection. In order to confirm the diagnosis, particularly when the illness is in its incubation or prodromal stages, laboratory testing is ultimately required. The main tools used in laboratory diagnostics are molecular biology and immunological detection techniques. Unfortunately, molecular assays for *T. haneyi* are few, and the nucleic acid detection methods available for the detection of *T. equi* are not able to detect *T. haneyi* infection. Although light microscopy examination of thin blood smears is regarded to be the traditional method for detecting and identifying all parasites, this method is difficult to perform in both acute cases and in inapparent infections. Meanwhile, the most widely available diagnostic assay, nPCR, can lead to false-negative results due to multiple factors [24]. The qPCR assay, which is an affordable method, has proven to be useful in the detection of other potential agents of EP, such as the *T. equi*/*B. caballi* group [25]. However, there is no qPCR method for the specific detection of *T. haneyi*, and most of the qPCR assays for detecting EP are based on 18S rRNA and are not able to differentiate *T. haneyi* from *T. equi*.

In this study, for the first time, we developed a qPCR assay based on the 2118 bp chr1sco of *T. haneyi* for the quantitative detection of *T. haneyi*. The chr1sco of *T. haneyi* is a specific sequence to *T. haneyi*, and differs from the sequences of other potential agents of the EP. We used this sequence as a detection target. TaqMan-MGB probes and primers were designed for the target sequence of *T. haneyi*. The diagnostic performance of the assay was evaluated comparatively with the optimized nPCR assay. The new qPCR assay’s specificity was evaluated using the other frequent infections of horses, while the sensitivity of the assay was evaluated using serially diluted plasmids. The reaction conditions were optimized, and the results were verified, and the newly developed qPCR has proven to be a reliable and convenient qPCR assay for the detection of *T. haneyi* in equine clinical specimens, with good sensitivity and specificity.

A number of detection systems are available for real-time PCR, including double-stranded DNA (dsDNA)-binding dyes and probe-based chemistry. Nonspecific dsDNA-binding dyes bind any dsDNA sequence and then fluoresce; thus, assays based on dsDNA-binding dyes need to ensure that the observed fluorescence amplification is not generated from unspecific amplification but rather only from the specific target sequence. The robust TaqMan chemistry, which is well-known as a rapid, specific and sensitive real-time PCR method, has many advantages. In our study, we used the specific TaqMan-MGB probe. TaqMan Minor Groove Binding (MGB) probes, an improvement over the standard TaqMan probe, have gained popularity recently due to the following benefits: based on the conventional TaqMan probe and TaqMan MGB changed the 3′ end non-fluorescence quenching group with MGB. The conjugated MGB probes are iso-helical and have a deep, narrow minor groove formed by the terminal 5–6 bp. The MGB embeds strongly into the double helix structure of templates and probes, making the differentiation of specific motifs possible. To improve the specificity of amplification products, the Tm value of a probe can be raised by around 10 °C without increasing the base number of the probe; for the same Tm value, a TaqMan MGB probe can have a shorter design than a regular TaqMan probe. This ensures that the fluorescent signals are specific to the target sequences and the probes can be directed at short specific regions [26]. Thus, our novel qPCR technique based on TaqMan-MGB probes might combine high specificity with exceptional sensitivity and reduce the fluorescent background [27]. The creation of primers and probes is now simpler and more reliable because of the advancement of TaqMan MGB probe labeling technology. This approach, which is more sensitive and specific than traditional RT-PCR and SYBR green, fluorescent RT-PCR, uses a specific primer pair and specific probe sequence to double-guarantee the specificity of detection [28,29]. As a result, our study effectively established a TaqMan-MGB qPCR test based on particular primers and a probe targeting the conserved region of the chr1sco gene of *T. haneyi*.

However, the qPCR assay does still have some limitations in this context. The Ct value cannot be directly interpreted as pathogenic load in many cases. The evaluation of the reliability and robustness of the standard curve is usually regarded as the key to accurately quantifying the expected copy number of pathogens [30]. However, the chr1sco of *T. haneyi*, which was chosen as the detection target, is a single-copy gene, meaning that samples with low pathogenic load might show high Ct values [10]. The Ct values of the same sample could fluctuate dramatically between tests due to variations in amplification efficiency and noise band adjustment, particularly for samples with low pathogenic loads [30]. And that is why this method showed the false-negative results. Therefore, it is critical to take into account other tactics that overcome the qPCR method’s drawbacks.

## 5. Conclusions

In conclusion, here for the first time, we developed a quantitative detection method for *T. haneyi* based on qPCR and validated the method by comparison with an optimized nPCR assay. The novel method showed high sensitivity and specificity. This study shows that the newly developed qPCR assay will provide a valuable tool for the rapid laboratory diagnostic assessment of *T. haneyi* infections in equids. Accordingly, the present study provides a precise quantitative assay and effectively reduces the infection of EP.

## Figures and Tables

**Table 1:** Primers and probes of the real-time PCR.

Primer Name	Primer Sequences (5′-3′)	Products
*T. haneyi-F:*	AATCCAAAACCAGCT	97 bp
*T. haneyi-R:*	GTACAAATCTCCCTAGAG	
*T. haneyi-Probe:*	FAM-CCTCCAAGTCGTCGT-MGB	

## Data Availability

Not applicable.
